# An Islamic Form of Logotherapy in the Treatment of Depression, Anxiety and Stress Symptoms in University Students in Iran

**DOI:** 10.1007/s10943-021-01495-0

**Published:** 2022-01-11

**Authors:** Shapour Fereydouni, Simon Forstmeier

**Affiliations:** 1 Developmental Psychology and Clinical Psychology of the Lifespan, Institute of Psychology, University of Siegen, Adolf-Reichwein-Str. 2a, 57068 Siegen, Germany; 2 Department of Educational Psychology, Islamic Azad University, Gachsaran Branch, Gachsaran, Iran

**Keywords:** Spiritually sensitive psychotherapy, Logotherapy, Depression, Anxiety, Stress, University students

## Abstract

Previous research demonstrated that spiritually sensitive psychotherapy is an effective treatment for clients with depression or anxiety, with outcomes equivalent to secular control interventions. The goal of this study was to evaluate the efficacy of spiritually sensitive logotherapy intervention in the treatment of depression, anxiety, and stress symptoms in university students in Iran. Sixty students with elevated depression symptoms (Beck Depression Inventory II, BDI-II, 22 or greater) were randomly assigned to either a twelve-session group logotherapy programme or a control group. Results showed that spiritually sensitive logotherapy significantly reduced depression, anxiety, and stress, and significantly more so than in the control group (e.g. interaction effect for BDI-II: *F* = 56.8, *p* < 0.001, with a large effect size).

## Introduction

Approximately, one in five individuals (17.6%) meets the criteria for a common mental disorder in any given year, and 29.2% at some time during their lives, as a recent meta-analysis concludes (Steel et al., 2014). Despite some regional variation in mental disorders, common mental disorders are highly prevalent globally, affecting people across all regions of the world. Depressive disorders (Ferrari et al., 2013), anxiety disorders (Baxter et al., 2013) and stress-related disorders (Kalia, 2002) are among the most frequent of these mental disorders. They contribute to the global burden of disease as they are associated with impaired physical and mental functioning, more work days lost, increased impairment at work and a high use of health care services (Vigo et al., 2016).

### Depression, Anxiety and Stress Symptoms in Iranian Adults and University Students

Mental health surveys in Iran have found similar prevalence rates (Noorbala et al., 2004). A fifth (21%) of the population had mental disorders; the most frequent symptoms were depressive (21%) and anxiety (20.8%) symptoms (Noorbala et al., 2004). Another study found a higher prevalence for depression (28.8%) than for anxiety disorders (14%) in Iranian adults (Alizadeh et al., 2019). Many studies on mental health in Iran use the Depression, Anxiety, and Stress Scale (DASS, Lovibond & Lovibond, 1995) as an assessment tool. On the DASS, the prevalence of depression, anxiety and stress has been found to be 29%, 32.2% and 34.8%, respectively, in an adult population in Iran (Mirzaei et al., 2019).

Younger people seem to be more likely to have a mental disorder than older people. College students in the USA exhibit a higher prevalence of depression (11.8%) than the general population (5.2%) (American College Health Association, 2007). The potential negative consequences of mental disorders in university students are poor academic performance, social problems, and increased rates of substance use and suicide. Despite the need for psychosocial help, many US students do not have access to any services (Eisenberg et al., 2007). Reasons for this were found to be lack of perceived need, being unaware of services or insurance coverage, scepticism about treatment effectiveness, low socioeconomic background and being Asian or a Pacific Islander. A considerable proportion of university students in Iran also exhibit elevated levels of depression, anxiety, and stress (Dehdari et al., 2013).

A meta-analysis on depression, anxiety and stress symptoms using the DASS in Iranian university students found higher mean values than comparable studies in other countries (Mohammadzadeh et al., 2019). This might be because mental disorders are even more stigmatising in this Muslim country than in Western countries, and this keeps students from seeking help (Taghva et al., 2017).

### Psychotherapeutic Services and Psychotherapy Research in Iran

Most of the psychotherapy approaches that have been developed in Western societies are also offered in Iran. In a representative sample of psychotherapists and psychiatrists in Iran, the most frequently used approach is cognitive behavioural therapy (CBT), with about 51% of all professionals using this approach (Mousavi et al., 2018). Psychoanalytic and psychodynamic approaches are used by about 11%, cognitive therapy is used by 11% and an integrated approach is used by about 11%.

Iranian researchers are also involved in psychotherapy research. Just a few recent examples are research on CBT and positive psychotherapy for depression (Asgharipoor et al., 2012), cognitive therapy and spiritual therapy for depression (Safara et al., 2012), schema therapy for depression (Hashemi & Darvishzadeh, 2016), mindfulness-based psychotherapy for psychological symptoms in opioid-dependent patients (Jenaabadi & Jahangir, 2017), metacognitive therapy for hypoactive sexual desire disorder (Ramezani et al., 2018) and spiritually supported psychotherapy for depressed cancer patients (Eilami et al., 2019).

### Logotherapy

Logotherapy is also one of the psychotherapeutic approaches that is offered and researched in Iran. Logotherapy was developed by Viktor Frankl (1962) and is based on the premise that everybody searches for meaning, and that an individual answer to the question of the meaning in life is the mechanism of change in psychotherapy.

There are several reviews of empirical research on the efficacy of logotherapy (Batthyány, 2011; Batthyány & Guttmann, 2006; Thir & Batthyány, 2016). The picture is clear: logotherapy decreases symptoms such as depression and increases meaning in life more than in control groups. For example, a six-session autobiographic logotherapy intervention was found to significantly reduce depressive symptoms and increase purpose in life when compared to results for a control group (Bernstein et al., 2012).

In Iran, there is an increasingly strong interest in logotherapy in university-based counselling and therapy centres. This makes sense from a developmental perspective: university students are in the midst of identity development, which includes a search for meaning and purpose in life. A logotherapy approach in this phase of life is supported by Erikson’s developmental theory of life stages (Erikson, 1959), in which he describes finding a solution to the identity crisis as the most important developmental task of adolescence and emerging adulthood. Students are regularly confronted with failures and successes not just in the academic realm, but also in social relationships. A failure to find purpose and meaning can lead to the development of a depressive disorder (Frankl, 1988). This is supported by Dehdari et al. (2013), who found that meaning in life was negatively associated with depression and anxiety in a sample of Iranian university students.

Psychotherapy research in Iran has investigated the efficacy of group logotherapy in university students at various places in the country. A randomised controlled trial (RCT) found that ten sessions of group logotherapy significantly reduced depressive symptoms and increased meaning in life when compared to results for an untreated control group (Robatmili et al., 2015). An eight-session group logotherapy course for female students with depression was shown to significantly increase psychological well-being and decrease addiction potential (Esalati et al., 2019). A logotherapy intervention for parents of difficult children led to a significant improvement in marital satisfaction when compared to results for a control group (Farahini et al., 2019).

It has been suggested that the group setting is particularly fruitful for exploring existential concerns in logotherapy because the individuals can benefit from the existentialism that is embedded in group processes. Yalom (2005) puts it this way: “The group configuration is not ‘you,’ the therapist, and ‘they,’ the dying; but it is we who are dying, we who are banding together in the face of our common condition” (p. 34).

### Spiritually Sensitive Psychotherapy (and Logotherapy)

Logotherapy (Frankl, 1962, 1988) can work without any reference to religiosity or spirituality (Crumbaugh, 1979). However, the individual’s way to find meaning in suffering and in life can include spiritual perspectives and activities (Okan & Eksi, 2017). Therefore, if spirituality is important to a particular patient, including the spiritual dimension in psychotherapy is a promising approach as tailoring treatments to the patient’s values improves efficacy (Sotsky et al., 1991). Religious patients usually want to discuss spiritual issues in psychotherapy (Rose et al., 2008).

A psychotherapeutic treatment that integrates religion and spirituality into psychotherapy has been called “spiritually sensitive psychotherapy” (Sperry, 2012), “religion-accommodative psychotherapy” (McCullough, 1999), and “faith-adapted psychotherapy” (Anderson et al., 2015). A review of the effects of religion-accommodative psychotherapy for depression and anxiety found a total of 11 articles published between 1992 and 2008 (by seven different research teams) (Paukert et al., 2011). Cognitive therapy (CT) was the secular control intervention in nine of those 11 studies. Six studies incorporated the intervention with a Christian perspective, and five with an Islamic perspective. The studies demonstrated that religion-accommodative psychotherapy was an effective treatment for clients with anxiety or depression, with outcomes equivalent, although not superior, to the control CT interventions. However, there was evidence to suggest that religion-accommodative cognitive therapy is more effective than secular CT for highly religious individuals.

A more recent meta-analysis found similar results for Christian, Muslim, Jewish and Taoist adaptations of cognitive behavioural therapy (CBT) (Anderson et al., 2015). The authors also analysed the methods of incorporating religion and spirituality into the treatment, which were combinations of the following:

“Discussion of religious teachings or scriptures as supportive evidence to counter irrational thoughts or to support cognitive or behavioural change;

Use of positive religious or spiritual coping techniques (for example, applying scriptural or spiritual solutions to the psychological problems of fear, anger, guilt, shame or despair);

Promotion of helpful belief or value systems, or use of shared value systems to strengthen therapeutic relationships;

Incorporation of religious practices such as prayer” (p. 187).

Logotherapy, as already noted above, can generally embrace spiritual issues because spirituality is one way to find meaning in life. However, spiritually sensitive (or spiritually oriented) logotherapy explicitly includes religious and spiritual issues (Okan & Eksi, 2017). This is also true for Islamic spirituality; as Okan and Eksi (2017) put it: “The basic philosophy of logotherapy is not far from Islam. However, Muslim psychotherapists have not addressed this issue in depth. Some religious and spiritual arguments have been placed among the logotherapeutic interventions in Iran, Pakistan, and several other Islamic countries” (p. 153).

Interestingly, most of the studies on logotherapy in Iran have applied a traditional rather than a spiritually adapted form of logotherapy (Esalati et al., 2019; Robatmili et al., 2015). Only Farahini et al. (2019) used a logotherapy programme “based on Islamic-Iranian values”, without giving details. Thus, the aim of the present study is to develop a therapy protocol based on logotherapy and incorporating Muslim values and practices.

### Aim of the Present Study

An investigation of spiritually sensitive logotherapy is needed because most logotherapy research in Iran, and also worldwide, has applied a more traditional version of logotherapy. Moreover, previous studies on logotherapy in Iran have only included participants with mild to moderate symptoms of depression and anxiety, and not severe symptoms indicating a major depressive disorder or an anxiety disorder. Furthermore, many studies investigating spiritually sensitive psychotherapy programmes use only small samples, such as ten, in each study group, and therefore their power to detect a medium effect was below 70% (Paukert et al., 2011).

Therefore, the aim of the present study is to evaluate the efficacy of a spiritually sensitive logotherapy programme that incorporates Muslim values and practices in alleviating the depression, anxiety and stress symptoms of university students with severe depressive symptoms.

## Methods

### Design

The present study is a randomised controlled study of individuals with elevated depression symptoms. The participants were randomised to either the logotherapy intervention group or the control condition group, which received a minimal, supportive intervention. All participants were assessed before (pretest) and after (post-test) the twelve weekly sessions.

Intervention group: the intervention group received twelve sessions of a spiritually sensitive logotherapy treatment. The sessions are described below in more detail.

Control group: the minimal intervention consisted of five sessions of supportive group discussions. Topics of discussion included sports, culture, jobs and successes. Control group discussions did not address the topics covered in the intervention group.

### Participants

#### Recruitment

The participants were recruited from two universities in Iran, Gachsaran University and Yasuj University and were all Muslims. A total of 830 students were screened for depressive symptoms using the Beck Depression Inventory II (BDI-II) (Beck et al., 1996). One hundred and twenty of them exhibited a elevated symptom level (total score of 22 or greater, see below) and were asked to participate in this study. Sixty individuals were included in the study and randomly allocated to one of the two study arms, after they had given their consent to participate.

#### Inclusion and Exclusion Criteria

Eligible participants were students at one of the participating universities, aged 18 or older, and having elevated depression symptoms as indicated by a BDI-II score of 22 or greater. The exclusion criteria were severe psychiatric comorbidity, acute suicidal ideations, and receiving an alternative psychological treatment during or less than 2 months prior to starting participation in the study.

#### Informed Consent

The objectives and goals, detailed information about assessment and intervention and the procedure of randomisation were explained to the participants. Written informed consent was obtained from all participants prior to inclusion. The study protocol was approved by the ethics committee of the Islamic Azad University of Gachsaran.

#### Sample Size

Previous studies on the efficacy of group psychotherapy for depression found large average effect sizes compared to no treatment (McDermut et al., 2001). As this is the first study on spiritually sensitive logotherapy, we base our sample size calculation on the conservative assumption of a moderate to large effect size for depression (*d* = 0.8, *f* = 0.4). The sample size was calculated using G*Power 3.1.0 software (Faul et al., 2007). Given an *α* = 0.05 and a test power of 1–*β* = 0.80, a total sample size of *n* = 40 is required to test the group x time interactions in an analysis of variance (ANOVA) with repeated measurements (*r* = 0.5). We assumed a rather conservative dropout rate of 30%, so we planned to enrol n = 60.

### Randomisation

After providing informed consent and receiving a baseline assessment, the participants were individually randomised. The allocation ratio for randomisation into either the intervention or control condition was 1:1. The randomisation was performed by a computer algorithm independently operated by the co-author at the University of Siegen, Germany.

### Spiritually Sensitive Logotherapy Intervention

#### Session Content and Setting

The spiritually sensitive logotherapy intervention consisted of twelve weekly group sessions, mostly 120 min in duration. Pretest and post-test assessments were conducted in additional sessions, so there were 14 sessions altogether. The sessions took place in rooms of the Islamic Azad University, Gachsaran Branch, Faculty of Nursing and Midwifery. Table 1 shows the content of all sessions.

**Table 1:** Description of sessions

No	Topic	Content of the sessions
1	Introduction	Examining the expectations of the participants; outlining therapeutic programme and session content; basic information about mental disorders, in particular depression, anxiety and stress
2	Positive memories	Aim: Keeping positive memories alive Theoretical background: Search for meaning (Frankl, 1962), concept of integration of experiences into biography (Erikson, 1959), life review and reminiscence (Pinquart & Forstmeier, 2012; Westerhof & Bohlmeijer, 2014) Therapeutic strategy: Sharing personal memories of pleasant religious events such as the birthday of Prophet Muhammad and the Shiite Imams
3	Cultural memories	Aim: Preserving positive cultural memories; using past experiences to confront the problems of the present time Theoretical background: Reminiscence (Pinquart & Forstmeier, 2012; Westerhof & Bohlmeijer, 2014) Therapeutic strategy: Sharing pleasant memories cultural events such as the Feast of New Year's Eve, the celebration of Charsanbeh Suri, etc. We used two short films for illustration (e.g. biography of Stephen Hawking, Frankl’s life in the concentration camp, visiting holy sites)
4	Personally significant memories	Aim: Review of personally significant memories in order to increase well-being Homework from the previous session: Thinking about personally significant memories (e.g. the birth of a sister or brother, marriage, etc.) and bringing notes to the session Theoretical background: Existentialism (Søren Kierkegaard) Therapeutic strategy: Sharing personally significant memories; reliving (imagining) the memories and focusing on positive emotions such as pleasure
5	Finding meaning in critical life events	Aim: Finding meaning in critical life events, suffering and hardship Homework from the previous session: Making a note of critical life events and suffering such as failure to complete lessons, love affairs, financial and educational problems, discomfort from a near-death experience, and then writing down religious or cultural ways of reducing or confronting suffering Theoretical background: Logotherapy (Frankl, 1962); problem-solving (D'Zurilla & Goldfried, 1971) Therapeutic strategy: Based on the examples from the homework, finding common and severe types of spirituality-related suffering (e.g. divine test by suffering and difficulty), or tolerance of suffering in culture and customs (e.g. boys learn from childhood that they should not be scared or cry); analysing problems and suffering with regard to their potential meaning
6	Meaning in life	Aim: Finding meaning in life Homework from the previous session: Thinking about your reason for living: why do you keep on living your life and not commit suicide? Theoretical background: Logotherapy; existential theory Throughout history, humanity has engaged with existential questions. Why are we here? For what purpose do we live in the world? Why are we standing here? What does life mean? (Okan & Eksi, 2017) Therapeutic strategy: Group discussion about potential answers to the question of the meaning of life; examining the purposefulness of life in accordance with the recommendations of religion and culture
7	Values	Aim: Discovering meanings and values and hope for life, and making an effort to live up to values Homework from the previous session: Thinking about the following question: What is valuable in life? Why is life worth living for you? Can a person live and die for his aspirations and values? Are you ready to sacrifice your life for your values? Theoretical background: Logotherapy; relationship between values and meaningfulness of life Therapeutic strategies: Two short films were presented, one on religious values and one on cultural values, and the answers were then discussed. Various values such as inherent cultural credentials were examined to find the relationship between meanings and values
8	Meaning of life in death	Aim: Finding meaning in death and nonexistence; hope and confidence Homework from the previous session: Thinking about the following questions: Does thinking about death make you feel hopeless or hopeful? Theoretical background: Logotherapy Therapeutic strategy: Presenting a film on the life of Steven Hawking, the physicist, and several other films on stories of people’s efforts to survive; discussing homework and looking for the meaning of life in death. Discussing the message that life is not eternal, therefore, we must make the most of it, and death is a part of life. Using religious resources to show that, with death, another life begins in heaven. Use of cultural heritage resources to argue that if we must die let it not be in bed but in struggle
9	Positive thoughts	Aim: Negative thoughts, positive thoughts, optimism and hope Theoretical background: Logotherapy; cognitive therapy (Beck et al., 1987) Therapeutic strategy: The session started with a brief reception with small pieces of chocolate. A half-full glass of water was then brought into the meeting and participants were asked whether it was half-full or half-empty. Several experimental and objective activities were also carried out. Afterwards, the subjects were asked to state if they had positive or negative thoughts,And how many times they had the negative thoughts each day. A few religious and cultural examples were provided. This session mostly used the cognitive therapy technique (to switch to positive thoughts instead of negative thoughts)
10	Freedom of will	Aim: Understanding the freedom of will, the meaning of love, responsibility, and morality Homework from the previous session: Thinking about the following questions: Do you have freedom in various different areas? Do you consider freedom to be free from restraints and obligations? Can you name different types of human responsibility? What is the meaning of love? What is the meaning of human morality? Theoretical background: Logotherapy; existential philosophy Therapeutic strategy: The homework was discussed, with a focus on the concept of freedom of will. A short film of hope and love of life was shown about a person who was perfectly successful with no hands and feet (Nick Vujicic, “Conquer the Peak of Success without Hands and Feet”)
11	Prayer as coping strategy	Aim: Investigating the effect of prayer as a coping strategy Homework from the previous session: Participating in Komeil Prayer on Friday. The prayer ceremony was part of the intervention programme Theoretical background: Theory of Frankl, various religious resources and a large body of research Therapeutic strategy: Discussing their experiences of praising god and praying; discussing the role of religious ceremonies in increasing well-being
12	Summary	Summarising the results of all therapeutic strategies and activities; evaluating the success of the intervention

#### Theoretical Background

The theoretical basis of this intervention is logotherapy by Frankl (1962). It has been adapted by the authors to include religious values and spiritual practices. Additional theoretical approaches were included in single sessions, namely Erikson’s life stages (Erikson, 1959), life review and reminiscence (Pinquart & Forstmeier, 2012; Westerhof & Bohlmeijer, 2014), and cognitive therapy (Beck et al., 1987).

#### Psychotherapist

The intervention was administered according to a manual by the first author, who is a trained psychotherapist and has a qualification in CBT and logotherapy. Supervision was administered by the second author.

### Outcome Measures

#### Beck Depression Inventory II (BDI-II)

The BDI-II is a 21-item self-report inventory that assesses symptoms of depression in the previous seven days (Beck et al., 1996). Each item is rated from 0 to 3 according to severity of difficulty experienced; total scores range from 0 to 63, with higher scores indicating more depression. The BDI-II has been shown to have good psychometric properties in psychiatric and non-psychiatric populations in various countries. We used the Persian (Farsi) translation of the BDI-II, which was developed by repeated translation and back-translation of the original questionnaire (Ghassemzadeh et al., 2005). In an Iranian student sample with 9.5% fulfilling the DSM-IV diagnosis of a Major Depressive Disorder (MDD), the BDI-II cut-off point of 22 or greater was the most suitable for screening MDD, whereas for screening milder but clinically significant depression, the cut-off point of 14 or greater was the most appropriate (Vasegh & Baradaran, 2014). Cronbach’s alpha was 0.70 in the present sample.

#### Depression, Anxiety and Stress Scale (DASS-42)

The DASS-42 is a 42-item self-report inventory that assesses symptoms of depression, anxiety and stress in the previous seven days (Lovibond & Lovibond, 1995). The depression subscale includes items evaluating symptoms such as anhedonia, sadness, worthlessness, hopelessness and lack of energy. The anxiety subscale includes items evaluating physiological arousal, phobias and situational anxiety. The stress subscale includes items evaluating symptoms such as difficulty in achieving relaxation, state of nervous tension, agitation, overreaction to situations, irritability and restlessness. Each item is rated from 0 to 3 according to the severity or frequency of the symptom. Each subscale has 14 items, and a participant’s score in each subscale is the sum of all items related to that subscale.

The DASS-42 has been shown to have good psychometric properties in psychiatric and non-psychiatric populations in various countries, and the three-factor structure has also been approved. We used the Persian (Farsi) translation of the DASS-42 (Afzali et al., 2007). Cronbach’s alpha was 0.91 for depression, 0.84 for anxiety and 0.84 for stress in the present sample.

### Statistical Analysis

All statistical analyses were performed using SPSS software, version 26 (SPSS Inc., Chicago, Illinois). Results were expressed as mean ± standard deviation (SD). Analyses of variance (ANOVAs) were conducted to compare the mean outcome in depressive, anxiety and stress symptoms. A significant interaction effect (group x time) is the criterion for the superiority of the intervention over the control group (between-group comparisons). Kolmogorov–Smirnov and Levene’s F test were used to evaluate the assumptions of normality of the variables and equality of variances, respectively. Statistical significance was determined as a p value of < 0.05. For the interpretation of Eta^2^, values over 0.01 indicate a small effect, over 0.06 a moderate effect, and over 0.14 a large effect (Cohen, 1992). In addition, effect sizes were reported as Cohen’s d. Within-effect sizes were computed as the difference between means of an outcome variable at the two time points divided by the pooled standard deviation for these means. Between-effect sizes were computed as difference of the differences between the means, divided by the pooled standard deviation at pretest. For the interpretation of Cohen’s d, values of 0.10–0.20 indicate a small effect, 0.40–0.60 a moderate effect, and over 0.80 a large effect (Cohen, 1992).

## Results

The mean age of the participants was 23.3 (SD = 2.26), and the range was between 20 and 27; there was no difference in age between the intervention and control groups. Gender distribution was 80% female and 20% male; there was also no difference here between the intervention and control groups. The demographic characteristics of the participants can be found in Table 2.

**Table 2:** A Comparison of Intervention and Control Group Characteristics at Baseline

Variables	Total	Logotherapy	Control	t/χ^2^	p
	M (SD)	M (SD)	M (SD)		
Age	22.3 (2.14)	22.4 (2.19)	22.1 (2.11)	−.54	.591
Gender (Female)	80%	83.3%	76.7%	.42	.519
Marital status					
Single	71.7%	66.7%	76.7%	.83	.661
Married/cohabitation	23.3%	26.7%	20.0%		
Separated/divorced	5.0%	6.7%	3.3%		
Depression (BDI-II)	31.6 (7.51)	31.2 (8.34)	32.1 (6.69)	.44	.659

**Fig. 1:**
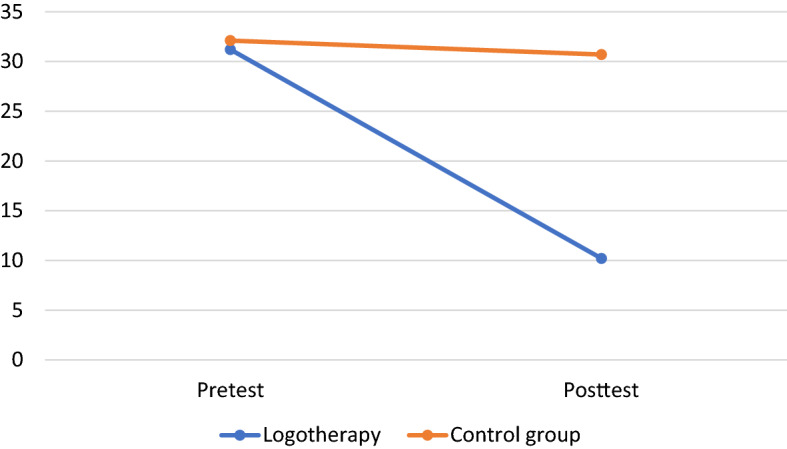
Depression (BDI-II) scores before and after the intervention

The mean and standard deviation of the scores obtained in the intervention and control groups as well as the results of the ANOVAs are presented in Table 3. The BDI-II scores are also presented in Fig. 1.

**Table 3:** Results of the two-way analyses of variance of the effect of the treatment

Variables	Group	Pretest	Post-test	Group effect^a^			Time effect^a^			Interaction effect^1^			Within	Between
		M (SD)	M (SD)	*F*	*P*	Eta^b^	*F*	*P*	Eta^b^	*F*	*p*	Eta^b^	Cohen’s d^2^	Cohen’s d^3^
Depression	Logotherapy	31.2 (8.3)	10.2 (7.1)	58.2	< .001	.50	73.3	< .001	.56	56.8	< .001	.50	2.72	2.55
(BDI-II)	Control	32.1 (6.7)	30.7 (7.5)										0.20	
Depression	Logotherapy	23.3 (4.03)	15.5 (1.3)	39.9	< .001	.41	37.9	< .001	.40	29.8	< .001	.34	2.61	1.76
(DASS)	Control	23.6 (4.08)	23.1 (3.9)										0.13	
Anxiety	Logotherapy	25.5 (2.47)	21.7 (2.0)	28.9	< .001	.33	26.2	< .001	.31	19.8	< .001	.25	1.69	1.51
(DASS)	Control	25.9 (2.04)	25.6 (2.1)										0.15	
Stress	Logotherapy	24.8 (3.8)	14.9 (1.5)	68.5	< .001	.54	59.9	< .001	.51	46.4	< .001	.44	3.43	2.40
(DASS)	Control	25.1 (3.79)	24.5 (4.2)										0.15	
Total Symptom Score	Logotherapy	73.6 (9.8)	52.1 (3.8)	55.4	< .001	.48	48.3	< .001	.45	37.4	< .001	.39	2.89	2.06
(DASS)	Control	74.6 (9.3)	73.2 (9.7)										0.15	

^a^ANOVA group, time, and interaction (group x time) effects

^b^“Within Cohen’s d” are within-effect sizes, computed as the difference between means of an outcome variable at the two time points divided by the pooled standard deviation for these means

^c^“Between Cohen’s d” are between-effect sizes, computed as difference of the differences between the means, divided by the pooled standard deviation at pretest

In general, the spiritually sensitive logotherapy intervention, adapted for culture and religion, significantly reduced depression, anxiety and stress, and significantly more than in the control group.

### Depression

The BDI-II scores of the logotherapy group decreased significantly more than those of the control group (interaction effect: *F* = 56.8, *p* < 0.001). The between-effect size was Cohen’s *d* = 2.55. Eta^2^ showed that the interaction effect could explain 50% of the variance of change in depression, which is a large effect. Homogeneity of variances was confirmed using Levene’s test, which showed that equal variances could be assumed in pretests (*F* = 0.29, *p* = 0.59) and post-tests (*F* = 0.22, *p* = 0.63).

With regard to DASS-42 depression scores, those of the logotherapy group decreased significantly more than those of the control group (interaction effect: *F* = 29.8, *p* < 0.001). The between-effect size was Cohen’s *d* = 1.76. Eta^2^ showed that the interaction effect could explain 34% of the variance of change in depression, which is a large effect. Levene’s test was non-significant in the pretests (*F* = 0.06, *p* = 0.93), but it was significant in the post-test (*F* = 19.48, *p* < 0.001), indicating the equality of error variances in the pretest, but the inequality of error variances in the post-test.

### Anxiety

The logotherapy group experienced a decrease in anxiety scores (DASS-42) that was significantly stronger than that in the control group (interaction effect: *F* = 19.8, *p* < 0.001). The between-effect size was Cohen’s *d* = 1.51. Eta^2^ showed that the interaction effect could explain 25% of the variance of change in anxiety, which is a large effect. Homogeneity of variances was confirmed using Levene’s test, which showed that equal variances could be assumed in the pretests (*F* = 0.57, *p* = 0.45) and post-tests (*F* = 0.07, *p* = 0.78).

### Stress

The logotherapy group experienced a more significant decrease in stress scores (DASS-42) than the control group (interaction effect: *F* = 46.4, *p* < 0.001). The between-effect size was Cohen’s *d* = 2.40. Eta^2^ showed that the interaction effect could explain 44% of the variance of change in anxiety, which is a large effect. Levene’s test was non-significant in both pretests (*F* = 0.007, *p* = 0.93), but was significant in the post-tests (*F* = 15.70, *p* = 0.0001), indicating equality of error variances in the pretest, but inequality of error variances in the post-test.

## Discussion

The results of the present study are very clear. The spiritually sensitive logotherapy intervention incorporating Muslim values and practices in university students with severe levels of depressive symptoms significantly reduced depression, anxiety, and stress, and significantly more so than in the control group. The effect sizes are large (Cohen, 1992). The improvements in the control group are very small, although control group members received a minimal intervention including five sessions with discussion about issues unrelated to those discussed in the intervention group such as sports, culture, jobs and successes.

The effect size is larger than that in previous studies on logotherapy with university students in Iran (Esalati et al., 2019; Farahini et al., 2019; Robatmili et al., 2015). There might be several reasons for this: (1) our programme incorporated spiritual values and practices that might meet the needs of the Muslim students; (2) there were twelve sessions, whereas in the previous studies, there were only eight or ten; (3) the sample consisted of students with severe depressive symptoms, in contrast to only mild to moderate levels in the other studies. Large symptom reduction is more likely when the pretest symptoms are severe.

The previous reviews and meta-analyses on “spiritually sensitive psychotherapy” (Sperry, 2012), “religion-accommodative psychotherapy” (McCullough, 1999; Paukert et al., 2011), and “faith-adapted psychotherapy” (Anderson et al., 2015) only included Muslim versions of CT or CBT, but not logotherapy. The present study complements these previous studies and adds to the knowledge of efficacious psychotherapies. Interestingly, the studies on Muslim CT and CBT programmes yielded only small to moderate effect sizes—in contrast to the large effect sizes we have found. There are various possible reasons for the smaller effect sizes found previously, including methodological weaknesses. In our view, more research involving the direct comparison of Muslim versions of CBT and logotherapy, with same number of sessions, is needed in order to have a fair comparison.

Over recent decades, the mental health care system in Iran has experienced some positive developments such as the establishment of health care centres in rural areas and an increase in mental health awareness (Danaei et al., 2019). However, a third of those with a diagnosis of a mental disorder still do not feel any need to access services and two thirds do not receive any health services for their mental problems (Rahimi-Movaghar et al., 2014). Insurance systems do not cover most non-pharmacological services for those with mental disorders (Danaei et al., 2019). Iranian mental health professionals are discussing which strategies are effective in reducing the stigma attached to mental disorders in Iran (Taghva et al., 2017). One answer to this question is the introduction of spiritually sensitive psychotherapy programmes at university counselling centres or general hospitals, because this would be in keeping with the values and interests of most Iranian citizens (Jafari et al., 2014; Hall & Powell, 2021).

Psychotherapists in Western countries need more “spiritual and religious competencies”, as Vieten et al. (2013) claim. On the basis of focus groups and surveys, they establish a set of 16 basic spiritual and religious competencies (attitudes, knowledge and skills) that all licensed psychologists should demonstrate in the domain of spiritual and religious beliefs and practices. For example, they propose that “psychologists inquire about spiritual and/or religious background, experience, practices, attitudes and beliefs as a standard part of understanding a client’s history” (p. 135). With regard to Muslim patients in Western countries, Hodge and Nadir (2008) suggest that psychotherapists “must unwrap the secular terminology used to express the underlying therapeutic precepts and then repackage the precepts in terminology that reflects Islamic teaching” (p. 31). We believe that the present study describes a concept that will be of help for psychotherapists.

### Study Limitations

Several limitations must be considered when interpreting the results. First, the sample consists of university students, thus the results are not generalisable to other age groups and other educational levels or to Muslim populations in general. Second, diagnostic interviews were not used for the diagnosis of depression, anxiety and stress-related disorders; participants were included on the basis of a self-report questionnaire (DASS-42) and cut-off values. Third, we did not conduct follow-up assessments, so no conclusions can be drawn about the long-term effect of the intervention. Fourth, only symptoms of depression, anxiety, and stress were investigated. Measures of meaning in life, spiritual well-being or other positive well-being variables have not been applied but would demonstrate the effectivity of the intervention on other psychological processes. Finally, measures of treatment fidelity were not used, thus, we cannot determine how closely the therapist followed the manual.

### Suggestions for Future Research

The present study can be the basis for future research on spiritually sensitive logotherapy for Muslim individuals. The treatment protocol has proven to be effective, with twelve weekly group sessions, mostly 120 min in duration. Future studies should use this treatment protocol and extend it with regard to several aspects. First, a follow-up assessment after three, six or twelve months should be implemented in order to investigate the long-term effect. Second, an active control group such as a CBT treatment, adapted for Muslim individuals, should be used to avoid the methodological weaknesses of previous studied on religion-accommodated CBT (Anderson et al., 2015). Third, this research should involve not only students, but different age groups and various sociodemographic backgrounds in Iran, in other Muslim countries, and with Muslims in Western countries.
